# Animal models for the study of ADHD: the need for next-generation models

**DOI:** 10.3389/fpsyt.2026.1773090

**Published:** 2026-03-25

**Authors:** Hiroki Furuie, Taichi Hatakeyama, Wakana Harigai, Taiichi Katayama, Kenji J. Tsuchiya, Nagahide Takahashi

**Affiliations:** 1 Department of Developmental Disorders, National Center of Neurology and Psychiatry, Kodaira, Japan; 2 Graduate School of Agricultural and Life Sciences, The University of Tokyo, Tokyo, Japan; 3 Research Fellowships for Young Scientists, Society for the Promotion of Science (JSPS), Chiyoda, Tokyo, Japan; 4 United Graduate School of Child Development, The University of Osaka, Kanazawa University, Hamamatsu University School of Medicine, Chiba University and University of Fukui, Suita, Japan; 5 Research Center for Child Mental Development, Hamamatsu University School of Medicine, Hamamatsu, Japan

**Keywords:** ADHD, animal model, behavior, environment, genetics

## Abstract

Animal models have long been indispensable for elucidating the pathophysiology of neurodevelopmental disorders, including attention-deficit/hyperactivity disorder (ADHD). However, many existing models inadequately capture the full spectrum of ADHD symptoms and their underlying neurobiological mechanisms. In this Mini Review, we argue that continued reliance on simplistic genetic models limits mechanistic insight and that next-generation approaches must integrate gene–environment (G×E) frameworks, together with behavioral readouts informed by the triple-pathway model of ADHD. Such approaches are essential for interrogating pharmacoresistant domains of ADHD, including executive dysfunction and impaired time perception, which remain inadequately addressed by current pharmacotherapies.

## 1 Introduction

ADHD (attention-deficit/hyperactivity disorder) is one of the most prevalent neurodevelopmental disorders, with an estimated prevalence of about 5% in children under 18 years of age (1, 2). Twin and family studies have shown that ADHD is highly heritable, with genetic factors accounting for up to 75% of the variance (3). Several environmental factors, including low birth weight, prematurity, maternal depression, and maternal immune activation, have also been implicated in the development of ADHD (4, 5). Given its high heritability, genetic approaches have been useful in generating animal models of ADHD to elucidate its underlying mechanisms.

Neurobiologically, ADHD has been associated with alterations in frontostriatal circuitry, particularly involving dopaminergic and noradrenergic signaling (6), as well as emerging evidence implicating glutamatergic transmission and synapse-related proteins (7, 8). Structural and functional imaging studies consistently report delayed cortical maturation and altered prefrontal development in individuals with ADHD (9). Current pharmacological treatments, including psychostimulants (e.g., methylphenidate, amphetamine) and non-stimulants (e.g., atomoxetine, guanfacine), primarily enhance catecholaminergic transmission in prefrontal networks, thereby improving attention and impulse control (10, 11). However, these treatments show limited efficacy for executive dysfunction and temporal processing deficits (12), highlighting the need for refined preclinical models with improved construct and predictive validity.

Although several genetic models have been developed, mechanistic understanding has lagged for several reasons. First, recent GWAS (genome-wide association studies) and epidemiological studies confirm that ADHD is a polygenic disorder rather than a single-gene disorder (8, 13). Although ADHD is strongly heritable, large-effect genetic variants are rare, suggesting that its genetic liability is not driven by single mutations in many cases. In this framework, ADHD risk reflects the additive influence of a large number of common variants, each contributing only a small increment to liability. Therefore, manipulation of a single gene in animals is considered to be insufficient to recapitulate the full features of ADHD-related changes in humans. Second, ADHD is not solely a genetic disease but results from interactions between genetic and environmental factors (3). In this framework, it has been proposed that genetic liabilities derived from common polymorphisms associated with ADHD could increase susceptibility to prenatal environmental perturbations, thereby contributing to developmental pathways associated with ADHD. Third, although ADHD is diagnosed based on hyperactive/impulsive and inattentive symptoms, the neural basis of ADHD is considered more complex. For example, the triple-pathway model is considered one of the most widely accepted theories of ADHD (14), yet few animal studies focus on these phenotypes. The triple-pathway model proposes that ADHD arises from dissociable neuropsychological pathways, including deficits in inhibitory control, delay-related processing, and temporal processing, extending the disorder beyond simple diagnostic symptom categories. However, most existing animal models have primarily focused on hyperactive/impulsive and inattentive symptoms, with relatively little attention to the cognitive domains implicated by the model. Current medications, including psychostimulants and non-stimulants, ameliorate some behavioral symptoms, particularly hyperactivity and impulsivity, but leave other domains, such as executive dysfunction and impaired time perception, which represent core components of the triple-pathway model, largely untreated (12, 15, 16). These symptoms are considered to contribute to substantial functional impairment in individuals with ADHD across the lifespan (17).

Importantly, ADHD is conceptualized as a neurodevelopmental disorder characterized by atypical trajectories of brain maturation rather than static neurochemical abnormalities (18). This developmental perspective underscores the importance of modeling early-life environmental exposures in interaction with genetic susceptibility.

Therefore, refined animal models that integrate genetic predispositions with environmental triggers, and that target neural dysfunctions observed in ADHD, are essential for advancing mechanistic insight and developing improved treatments. Here, we first summarize currently available animal models for ADHD, classified by hypothesis (genetic models and environmental models) and discuss the utility of refined gene- environmental interaction models. We also summarize the readouts used in the animal studies of ADHD and discuss the importance of focusing not only on diagnostic symptoms but also on neural dysfunction of ADHD, to gain deeper insight into the mechanism and potential interventions for pharmacoresistant features of ADHD.

This narrative review is based on a structured but non-systematic literature search conducted primarily in PubMed. We focus on experimental rodent models relevant to ADHD, including genetic, environmental, and gene × environment paradigms, as well as studies addressing behavioral readouts and pharmacological validation. Priority is given to peer-reviewed articles and recent large-scale genetic and epidemiological findings.

## 2 Current animal models of ADHD

### 2.1 Genetic models of ADHD

Several genetic models have been proposed to investigate the neurobiological mechanisms of ADHD. Representative examples and their key characteristics are summarized in **Table 1**.

**Table 1:** Genetic models of ADHD.

Model	Genetic basis	Behavioral phenotypes	Neurochemical features	Strengths	Limitations	Key reference
DAT-KO Mouse/Rat	Knockout of dopamine transporter (*Slc6a3*)	Hyperactivity, impulsivity, working memory deficits	Extreme hyperdopaminergia; psychostimulant effect via serotonergic pathways	Strong face validity; robust and reproducible hyperactivity	Poor construct validity (dopamine profile opposite to human ADHD); limited predictive validity	(19–23)
DRD4 KO/Transgenic Mouse	Deletion of *Drd4* or knock-in of human VNTR variants	Increased novelty seeking, altered locomotor activity, attention deficits	Altered dopaminergic signaling, particularly in prefrontal cortex and striatum	Provided early mechanistic insights into dopaminergic contributions; linked to candidate gene era findings	DRD4 7-repeat VNTR no longer supported as a robust ADHD risk locus in GWAS; limited translational relevance	(24, 25)
Coloboma Mouse	SNAP-25 mutation	Hyperactivity, impulsivity, cognitive deficits	Dysregulated dopaminergic and noradrenergic systems	Models treatment-resistant subgroup (responds to amphetamine, not methylphenidate)	Limited construct validity; rare mutation not common in human ADHD	(26, 27)
Lphn3 KO (Mouse/Rat)	Latrophilin-3 deletion (candidate ADHD risk gene)	Hyperactivity, impulsivity, learning deficits	Altered dopamine synthesis and receptor expression	Historically replicated in candidate studies; heuristic value	Not confirmed in large GWAS; behavioral spectrum not fully characterized	(28, 29)
mGluR5 KO/Antagonist Models	Targeted deletion or pharmacological inhibition of mGluR5	Hyperactivity, impaired reinforcement learning, cognitive inflexibility	Altered glutamatergic signaling; disrupted synaptic plasticity	Strong link to glutamatergic pathways and CNV studies; translational relevance	Less validated in ADHD context; phenotypes depend on assay	(30–33)
SHR Rat	Selectively bred for hypertension	Hyperactivity, inattention, impulsivity	Frontostriatal dopaminergic dysregulation; altered noradrenergic function	Widely used; exhibits all three core behavioral traits	Hypertension as a confound; WKY control strain problematic	(34, 35)
Lister Hooded (LH) Rat	Outbred strain used as ADHD-like model	Hyperactivity, inattention, impulsivity; improvement with atomoxetine	Differences in gut microbiota; trend toward increased dopamine in prefrontal cortex after FMT	ADHD-like behaviors validated pharmacologically; used in microbiota–brain axis studies	Still limited replication; model adoption remains preliminary	(36, 37)

Summary of representative rodent genetic models of ADHD, including their genetic basis, behavioral phenotypes, neurochemical characteristics, and translational relevance.

ADHD, attention-deficit/hyperactivity disorder; DAT-KO, dopamine transporter knockout; DRD4, dopamine D4 receptor; Lphn3, latrophilin-3; mGluR5, metabotropic glutamate receptor 5; SHR, spontaneously hypertensive rat; LH, Lister Hooded (rat); DA, dopamine; NE, norepinephrine.

#### 2.1.1 Dopamine transporter knockout mice

DAT-KO mice display pronounced hyperdopaminergia, spontaneous hyperactivity, working memory deficits, and impulsive behavior. Although this model demonstrates strong face and construct validity, its hyperdopaminergic state differs from the hypo- or normodopaminergic profiles typically observed in patients (19). Moreover, psychostimulants attenuate hyperactivity in DAT-KO mice primarily through serotonergic rather than dopaminergic mechanisms (20). DAT1 was long considered a candidate risk gene for ADHD, but subsequent large-scale GWAS have not confirmed it as a genome-wide significant locus (8, 13).

More recently, DAT-KO rats have been developed and extensively characterized. Similar to DAT-KO mice, DAT-KO rats exhibit robust spontaneous hyperactivity, deficits in sensorimotor gating, working memory impairments, and pronounced impulsivity (21–23), supporting strong face validity as an ADHD-related model. These animals also display an exaggerated hyperdopaminergic phenotype (21). Despite the theoretical advantages of rat-based models for assessing higher-order cognitive functions, key paradigms for evaluating sustained attention and response inhibition, such as the five-choice serial reaction time task (5-CSRTT), have not been systematically applied to DAT-KO rats, leaving their cognitive phenotype incompletely characterized. Moreover, psychostimulants such as amphetamine and methylphenidate do not normalize behavioral phenotypes in DAT-KO rats, although partial reductions in hyperactivity have been reported (21–23). Taken together, these findings indicate that DAT-KO rats do not robustly fulfill predictive validity as a model of ADHD.

#### 2.1.2 DRD4 knockout and transgenic models

Dopamine D4 receptor (DRD4) knockout mice, as well as transgenic mice carrying human DRD4 variants, have been investigated as potential ADHD models. These mice exhibit increased novelty seeking, altered locomotor activity, and deficits in attention-related tasks, phenotypes that were considered relevant to the behavioral profile of ADHD (24, 25). Early genetic association studies, particularly those implicating the 7-repeat VNTR allele of DRD4, motivated the development of these models. However, recent genome-wide association studies have not confirmed DRD4 as a robust ADHD risk locus (8, 13). Thus, while DRD4 models retain heuristic value for studying dopaminergic contributions to attention and impulsivity, their direct translational relevance to ADHD genetics is limited.

#### 2.1.3 Coloboma mice (SNAP-25 mutation)

These mice exhibit hyperactivity, impulsivity, and cognitive deficits (26, 27). Both dopaminergic and noradrenergic systems are dysregulated. Notably, their hyperactivity responds to amphetamine but not to methylphenidate, resembling treatment-resistant ADHD subgroups. SNAP-25 mutations were suggested as ADHD risk variants in early studies, but they have not been supported by modern GWAS.

#### 2.1.4 Lphn3 knockout models

Latrophilin-3 knockout (Lphn3 KO) rats and mice exhibit hyperactivity, impulsivity, and learning deficits, along with alterations in dopamine synthesis and receptor expression (28, 29). Early human genetic studies reported replicated associations between LPHN3 variants and ADHD, and this gene was considered one of the most promising candidate loci. However, more recent large-scale GWAS meta-analyses have not confirmed LPHN3 as a genome-wide significant risk locus (8, 13). Thus, while Lphn3 KO models remain useful for investigating dopaminergic regulation of behavior, their translational relevance to ADHD should be interpreted with caution in light of current genetic evidence.

#### 2.1.5 mGluR5 knockout and pharmacological models

Metabotropic glutamate receptor 5 (mGluR5) has emerged as a candidate pathway in ADHD, supported by genome-wide copy number variation studies that implicate glutamatergic networks in the disorder (7). Mouse models with targeted deletion of mGluR5 (30, 31), as well as those exposed to pharmacological antagonists such as MPEP, exhibit hyperactivity, impaired reinforcement learning, and cognitive inflexibility (32, 33). These behavioral phenotypes align with deficits in reward processing and executive function observed in ADHD. At the neurochemical level, these models demonstrate disrupted glutamatergic signaling and altered synaptic plasticity, particularly within the prefrontal cortex and striatum. Although less extensively validated than dopaminergic models, mGluR5-based models provide translational relevance by linking rodent phenotypes to human genetic findings.

#### 2.1.6 Spontaneously hypertensive rat

The spontaneously hypertensive rat (SHR) is the most widely used ADHD model and exhibits all three core behavioral traits: hyperactivity, inattention, and impulsivity (34, 35). Although SHRs are not pure genetic models for ADHD, they demonstrate frontostriatal dopaminergic dysregulation and altered noradrenergic function. However, hypertension constitutes a major confounding factor, and the appropriateness of Wistar Kyoto (WKY) rats as controls remains debated. Unlike single-gene knockouts, SHR is a polygenic strain, which aligns better with current GWAS findings, although its genetic architecture remains incompletely mapped.

#### 2.1.7 Lister hooded rats

The Lister Hooded rats have recently been proposed as an ADHD model. Similar to SHRs, LH rats are not pure genetic models for ADHD, however, previous studies have reported that LH rats display hyperactivity, inattention, and impulsivity, and that these behavioral abnormalities are ameliorated by the ADHD drug atomoxetine (36). In addition, LH rats differ from Wistar rats in gut microbiota composition and show increased hyperactivity and impulsivity, supporting their face validity as an ADHD-like strain (37). Fecal microbiota transplantation (FMT) from healthy donors reduced hyperactivity in LH rats and tended to increase dopamine levels in the prelimbic cortex (37). These findings suggest that LH rats may serve as a promising complementary model for ADHD, particularly in studies addressing the interaction between microbiota, dopaminergic signaling, and behavior. However, independent replication studies remain limited, and their widespread adoption remains preliminary.

It should be noted that several of the genetic models described above exhibit behavioral and neurobiological phenotypes that overlap with other neurodevelopmental or psychiatric conditions, including schizophrenia-related models and mood disorder paradigms. Rather than representing disorder-specific entities, these models are better conceptualized as tools to investigate transdiagnostic behavioral dimensions—such as impulsivity, attentional control, or reward processing—that are relevant to ADHD. Therefore, construct validity should be evaluated based on domain-specific phenotypes and neurobiological signatures rather than categorical diagnostic boundaries (38, 39).

### 2.2 Environmental models

Epidemiological studies indicate that, in addition to strong genetic influences, perinatal environmental risk factors also contribute to ADHD. Such factors are estimated to account for 10–30% of the variance in ADHD liability. In light of these findings, a variety of rodent models has been developed to examine environmental risk factors implicated in ADHD. Representative environmental models relevant to ADHD are summarized in **Table 2**. These include chemical exposures (prenatal nicotine, lead, polychlorinated biphenyls [PCBs]) (39), maternal stress paradigms (prenatal restraint or variable stress) (40), nutritional manipulations such as perinatal zinc deficiency (41), and perinatal stress or pharmacological interventions [e.g., neonatal isolation (42), neonatal NMDA receptor blockade (43)]. While these models often lack neurochemical specificity and their behavioral outcomes can be inconsistent, they remain valuable for exploring modifiable risk factors and provide complementary perspectives to genetic models. Across these environmental models, offspring commonly exhibit behavioral phenotypes relevant to ADHD, including increased locomotor activity, attentional deficits, and impairments in executive function.

**Table 2:** Environmental models of ADHD.

Category	Example manipulation	Behavioral phenotypes	Neurobiological features	Key references
Chemical exposures	Prenatal nicotine, lead, manganese, cadmium, PCBs	Hyperactivity, attentional deficits, impulsivity	Altered catecholamine and glutamate signaling	(39, 44, 46–54)
Maternal stress	Prenatal restraint stress, variable stress paradigms	Hyperactivity, anxiety, impaired cognition	Dysregulated HPA axis, DA/NE imbalance	(40)
Nutritional factors	Perinatal zinc deficiency	Hyperactivity, inattention, impaired learning	Disrupted dopamine signaling; impaired synaptic plasticity	(41)
Perinatal stress/pharmacological insults	Neonatal isolation, neonatal NMDA receptor blockade	Working memory deficits, hyperactivity, altered sociability	Reduced NMDA receptor function, abnormal cortical maturation	(42, 43)
Low birth weight/prematurity	Maternal undernutrition, intrauterine growth restriction (IUGR), neonatal hypoxia	Hyperactivity, attentional deficits, executive dysfunction	Altered cortical development, impaired monoaminergic signaling	(55–64)

Environmental manipulations used to model ADHD-related phenotypes in rodents, including chemical exposures, maternal stress paradigms, nutritional risks, pharmacological interventions, and perinatal complications.

IUGR, intrauterine growth restriction; PCB, polychlorinated biphenyl; HPA axis, hypothalamic–pituitary–adrenal axis; DA, dopamine; NE, norepinephrine; NMDA, N-methyl-D-aspartate (receptor); CNS, central nervous system.

Among chemical exposure models, developmental exposure to heavy metals has received increasing attention. In addition to lead, cadmium and manganese have been modeled in rodents as neurodevelopmental toxicants relevant to ADHD-related phenotypes. Developmental manganese exposure, particularly during the postnatal period up to weaning, consistently induces long-lasting impairments in sustained attention, impulse control, and sensorimotor function, whereas effects on locomotor activity are inconsistent (44–47). Pharmacological studies indicate that methylphenidate attenuates impulsive responding in manganese-exposed animals but fails to rescue deficits in selective or focused attention, highlighting limited and domain-specific efficacy (44, 48). In contrast, developmental lead exposure induces impairments in learning and memory, attentional deficits, and increased impulsivity, while effects on hyperactivity are less consistent (47, 49, 50). The effects of ADHD medications on lead-induced cognitive deficits remain largely unexplored. Cadmium exposure during prenatal or early postnatal periods has been associated with learning and memory impairments and, in some studies, altered exploratory or locomotor behavior; however, ADHD-like phenotypes have not been fully characterized, and systematic pharmacological validation remains limited (51–54).

In addition to chemical, nutritional, and stress paradigms, perinatal complications have also been modeled in rodents. Low birth weight and prematurity-related models, such as intrauterine growth restriction (IUGR) (55), maternal undernutrition (56), or perinatal hypoxia (57–59), are used to mimic epidemiologically validated risk factors for ADHD. Among these, perinatal hypoxia represents a particularly well-characterized and clinically relevant environmental risk factor. Epidemiological studies link perinatal hypoxia and related obstetric complications to an increased risk of ADHD (60). To model these risk factors, rat models employing hypoxia or hypoxia/ischemia during prenatal, perinatal, or early neonatal periods have been developed. Such manipulations can produce long-lasting behavioral alterations, including hyperactivity, attentional deficits, impulsivity, and impaired cognition, partially overlapping with core ADHD symptoms (57, 61). However, behavioral outcomes are highly variable and depend on the timing and severity of exposure, with some models producing broader cognitive or motor impairments (59). Pharmacological validation has yielded mixed results, as psychostimulants do not consistently normalize hypoxia-induced behavioral abnormalities (62–65). Thus, these models capture clinically relevant environmental risk factors for ADHD but show limited specificity as standalone models.

Notably, environmental manipulations alone are insufficient to explain the substantial individual variability in outcomes, even when the same insult is applied. For this reason, models that incorporate gene × environment interactions represent an important next step toward addressing the complexity of ADHD.

Similarly, stress-related environmental paradigms may model behavioral dimensions shared with depression or anxiety disorders (40). This transdiagnostic overlap highlights the importance of carefully defining the behavioral and neurobiological features that justify their relevance to ADHD research.

### 2.3 Gene × environment interaction models

Few early studies attempted to combine genetic vulnerability with environmental triggers in rodent models, for example:

- Lphn3 knock out rat exposed to deltamethrin (66)
- Pax6 mutant with paternal aging (67)
- SHR exposed to juvenile chronic mild stress (68)
- SNAP25 knock out mouse exposed to prenatal nicotine exposure (69)

These approaches were valuable in highlighting the importance of gene–environment interactions in ADHD. However, with the advent of large-scale GWAS and meta-analyses, many of these candidate variants are no longer considered robust ADHD risk alleles (8, 13). This underscores the need to move beyond single-gene candidate models and instead develop interaction paradigms that better reflect polygenic liability and convergent biological pathways identified through contemporary genetics. Such next-generation G×E models may better capture the multifactorial nature of ADHD and provide more reliable platforms for studying comorbidity and treatment resistance.

## 3 Future directions

### 3.1 Prioritize gene × environment models

Future animal models should prioritize the integration of polygenic susceptibility with relevant environmental triggers. As discussed above, SHR rat is a well-validated polygenic model of ADHD-like traits, reflecting the cumulative effects of multiple genetic variants (3). On the other hand, epidemiological studies indicate that prenatal infection increases the risk of ADHD (70), highlighting perinatal inflammation as a relevant environmental factor. Maternal immune activation (MIA) has been widely used in the study of other psychiatric and neurodevelopmental disorders (71).

Importantly, accumulating evidence suggests that prenatal inflammatory exposure can alter cortical maturation, microglial activation, and long-term dopaminergic and glutamatergic signaling—processes that are highly relevant to ADHD pathophysiology (72). Neuroinflammatory perturbations during critical developmental windows may disrupt prefrontal circuit development and executive function (71–73), thereby providing a biologically plausible pathway linking perinatal inflammation to attentional and behavioral dysregulation.

Thus, one promising approach is to combine SHR with MIA, thereby modeling the interaction between genetic predisposition and perinatal inflammation (4). Such paradigms may more accurately recapitulate the multifactorial etiology of ADHD and allow investigation of comorbidities and treatment resistance (4, 71).

### 3.2 Expand behavioral readouts

**Figure 1:**
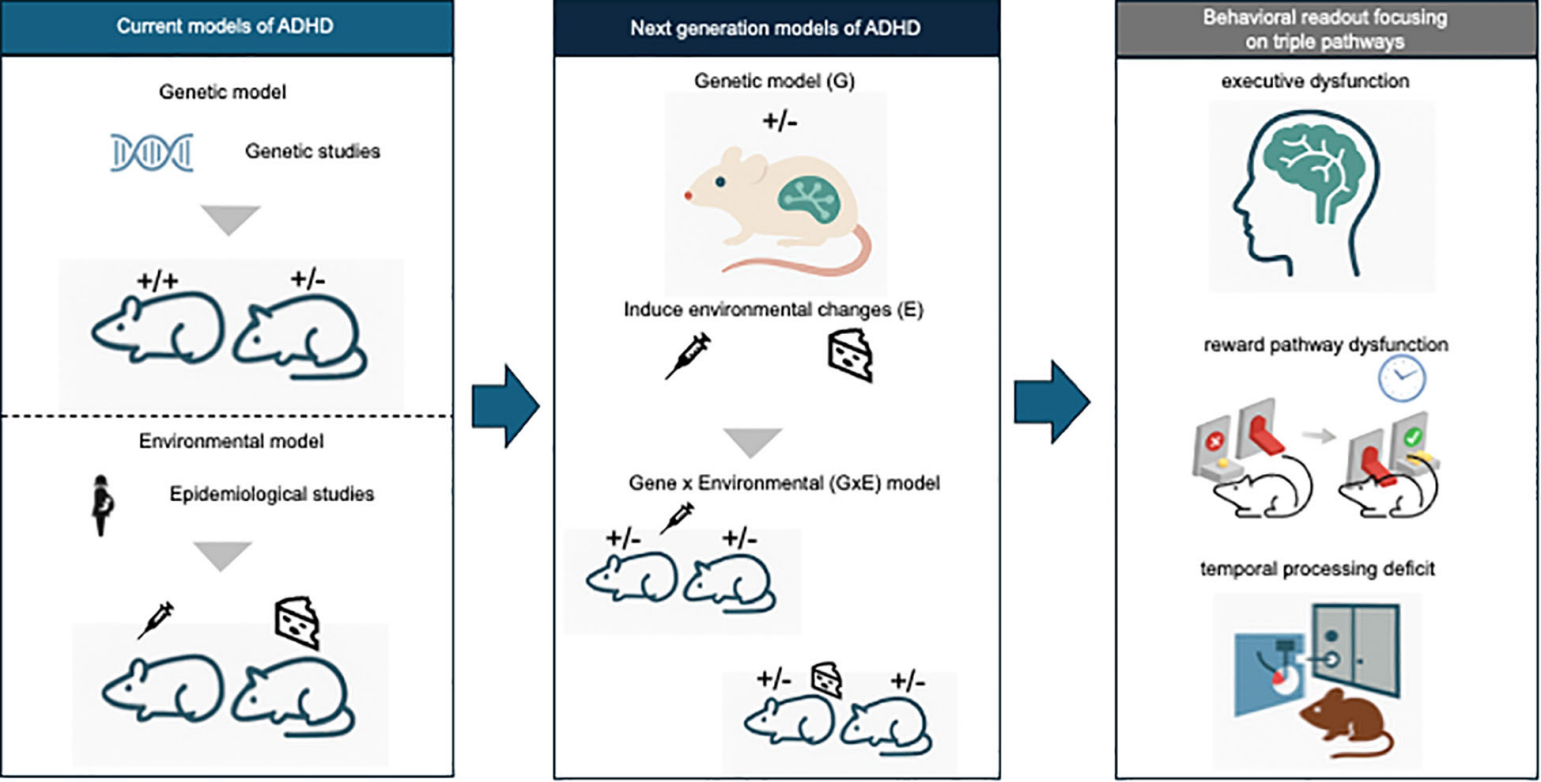
Next-generation animal models of attention-deficit/hyperactivity disorder (ADHD). **Figure 1** illustrates a conceptual framework for developing next-generation animal models of attention-deficit/hyperactivity disorder (ADHD). Left panel: Current models of ADHD, such as genetic models from results of genetic studies and environmental models from results of epidemiological studies. Middle panel: Next-generation models of ADHD which environmental perturbations are experimentally introduced into rodents with genetic susceptibility, enabling the study of gene × environment (G×E) interactions. Right panel: Behavioral assessments target neuropsychological domains emphasized by the triple-pathway model—executive dysfunction, reward pathway dysfunction, and temporal processing deficit—providing translationally relevant readouts for phenotypes that are often resistant to current pharmacological treatments.

Although ADHD is clinically defined by hyperactive, impulsive, and inattentive symptoms, these behaviors are thought to arise from disruptions in multiple neuropsychological domains. The triple-pathway model proposes that impairments in executive function, reward pathway–related processes, and temporal processing contribute to ADHD symptoms. However, traditional assessments have largely focused on locomotor hyperactivity. In line with the conceptual framework illustrated in **Figure 1**, next-generation models should incorporate behavioral tasks that systematically assess these core neuropsychological alterations through paradigms assessing sustained attention, impulsivity, and delay-related decision making, and prefrontal-dependent executive functions, such as:

▪ Temporal bisection tasks for time perception,
▪ Five-choice serial reaction time task (5-CSRTT) for sustained attention,
▪ Delay discounting or differential reinforcement of low-rate (DRL) schedules for impulsivity control,
▪ Temporal order recognition memory (TORM) for prefrontal-dependent executive function which provide complementary insight into prefrontal-dependent executive function.

Together, these tasks provide greater translational value by targeting higher-order cognitive dysfunctions that are central to ADHD pathophysiology and frequently insufficiently captured by conventional locomotor measures (see **Supplementary Table 1**).

### 3.3 Address pharmacoresistant symptoms

Current pharmacological treatments for ADHD are effective for hyperactivity and impulsivity but show limited efficacy for executive dysfunctions such as impaired time perception and planning. Next-generation animal models should therefore be explicitly designed to elucidate the neurobiological mechanisms underlying these pharmacoresistant domains. A deeper mechanistic understanding may open avenues for developing targeted pharmacological and non-pharmacological interventions that address the full spectrum of ADHD-related impairments.

### 3.4 Emerging tools

Rather than attempting to reproduce ADHD by introducing dozens of individual GWAS variants, which would be neither feasible nor conceptually accurate for a polygenic disorder, next-generation models should aim to emulate polygenic liability at the pathway and regulatory level. This can be achieved by targeting convergent biological processes highlighted by GWAS (8, 13) and CNV (7) studies, such as glutamatergic signaling, dopaminergic regulation, and neurodevelopmental transcriptional networks.

One illustrative example is to focus on key molecules that converge on ADHD-relevant pathways. Loss of progranulin (PGRN, encoded by GRN) can be conceptualized as simultaneously disrupting multiple pathways implicated in ADHD. PGRN deficiency promotes uncontrolled microglial activation and inflammation, compromises neuronal survival and axonal outgrowth in the prefrontal cortex, and impairs lysosomal homeostasis critical for synaptic plasticity (74–76). Notably, PGRN also contributes to glutamatergic synapse maturation (77, 78), linking it to NMDA/AMPAR pathways repeatedly highlighted in ADHD GWAS pathway analyses (8, 13). A cortical morphology GWAS reported an SNP (rs2696555) in the promoter of GRN associated with orbitofrontal and ventral frontal structure (79) highlighting a potential link between prefrontal development, inflammation, and executive dysfunction relevant to ADHD. Thus, disrupting PGRN function can be viewed as simultaneously perturbing inflammation, prefrontal dysfunction, and glutamatergic signaling—three central domains consistently implicated in ADHD pathophysiology.

Another potential emerging experimental tool is disruption of PACAP (pituitary adenylate cyclase-activating polypeptide, or ADCYAP1, adenylate cyclase activating polypeptide 1) signaling. Although direct human genetic evidence linking ADCYAP1 to ADHD remains limited, PACAP plays a critical role in neurodevelopment, catecholaminergic modulation, and prefrontal circuit function. PACAP-deficient mice exhibit hyperactivity, impaired sensorimotor gating, and responsiveness to atomoxetine, phenotypes relevant to ADHD (80, 81). From a pathway-based perspective, PACAP disruption can be conceptualized as a means to simultaneously perturb multiple biological domains repeatedly implicated in ADHD GWAS, including neurodevelopmental regulation and monoaminergic signaling. Thus, rather than serving as a classical genetic ADHD model, PACAP-based models may function as experimental tools to probe convergent ADHD-relevant pathways.

## 4 Conclusion

No single animal model can fully capture the multifaceted nature of ADHD, a disorder shaped by both polygenic risk and environmental influences (3). Nevertheless, integrating these components into animal models will bring us closer to understanding the neurobiological underpinnings of ADHD, particularly with respect to pharmacoresistant symptoms. Developing next-generation G×E models with rigorous behavioral, genetic, and pharmacological validation is essential for advancing the field (**Figure 1**).
