# Supplementary material for: Animal models for the study of ADHD: the need for next-generation models
Source: Front Psychiatry. 2026 Mar 25;17:1773090. doi: 10.3389/fpsyt.2026.1773090 (PMC13056871; doi:10.3389/fpsyt.2026.1773090)
Supplement: Supplementary file 1 [file Table1.docx]

Supplementary Material

# Supplementary Table1. Cognitive Tasks useful in ADHD Models

| **Task** | **Triple Pathway Domain** | **Cognitive Process Assessed** | **Neural Substrate** | **ADHD Relevance** | **Example in Models** |
| --- | --- | --- | --- | --- | --- |
| **Temporal Bisection** | Temporal processing deficit | Time perception | Striatum, cerebellum | Patients show impaired time estimation | Applied in rodent operant chambers |
| **5-CSRTT** | Executive dysfunction | Sustained attention, response control | Prefrontal cortex, basal forebrain | Sensitive to stimulant effects; parallels CPT in humans | Widely used in SHR and transgenic mice |
| **Delay Discounting / DRL** | Reward pathway dysfunction | Impulsivity, reinforcement sensitivity | Orbitofrontal cortex, striatum | ADHD patients prefer smaller immediate rewards | Used in SHR, DAT-KO |
| **TORM** | Executive dysfunction | Temporal order memory (working memory) | Prefrontal cortex (PFC) | Deficits in working memory and EF in ADHD; PFC-related | Deficits shown in SHR, rescued by MPH |
| **Reversal Learning / Set-Shifting** | Executive dysfunction | Cognitive flexibility, set-shifting | Orbitofrontal and medial PFC | Reflects executive dysfunction seen in ADHD | Emerging use in Lphn3 KO |

This table summarizes representative behavioral paradigms used to assess neuropsychological domains relevant to ADHD. Tasks are categorized according to the triple-pathway model—executive dysfunction, reward pathway dysfunction, and temporal processing deficits—and include their cognitive targets, underlying neural substrates, clinical relevance to ADHD, and examples of their application in established genetic and environmental models.

Abbreviations:

ADHD, attention-deficit/hyperactivity disorder; 5-CSRTT, five-choice serial reaction time task; DRL, differential reinforcement of low-rate responding; DAT-KO, dopamine transporter knockout; TORM, temporal order recognition memory; PFC, prefrontal cortex; MPH, methylphenidate; SHR, spontaneously hypertensive rat; Lphn3 KO, latrophilin-3 knockout.

**
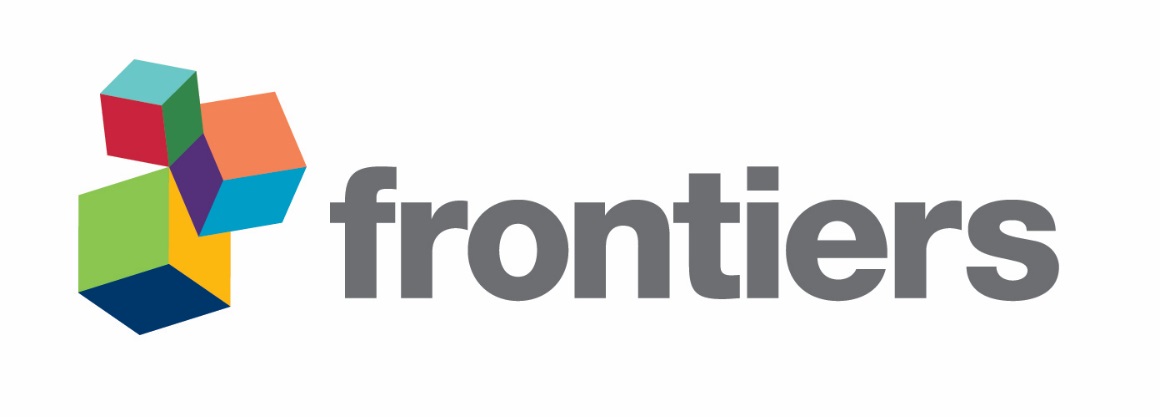
**
